# An analysis of three levels of scaled-up coverage for 28 interventions to avert stillbirths and maternal, newborn and child mortality in 27 countries in Latin America and the Caribbean with the Lives Saved Tool (LiST)

**DOI:** 10.1186/s12889-016-3238-z

**Published:** 2016-07-22

**Authors:** Lauren Arnesen, Thomas O’Connell, Luisa Brumana, Pablo Durán

**Affiliations:** Pan American Health Organization, Centro Latinoamericano de Perinatología, Salud de la Mujer y Reproductiva, Montevideo, Uruguay; UNICEF, New York Headquarters, New York, USA; UNICEF, Regional Office for Latin America and the Caribbean, Panama, Panama

## Abstract

**Background:**

Action to avert maternal and child mortality was propelled by the Millennium Development Goals (MDGs) in 2000. The Latin American and Caribbean (LAC) region has shown promise in achieving the MDGs in many countries, but preventable maternal, neonatal and child mortality persist. Furthermore, preventable stillbirths are occurring in large numbers in the region. While an effective set of maternal, newborn and child health (MNCH) interventions have been identified, they have not been brought to scale across LAC.

**Methods:**

Baseline data for select MNCH interventions for 27 LAC countries that are included in the Lives Saved Tool (LiST) were verified and updated with survey data. Three LiST projections were built for each country: baseline, MDG-focused, and All Included, each scaling up a progressively larger set of interventions for 2015 - 2030. Impact was assessed for 2015 - 2035, comparing annual and total lives saved, as projected by LiST.

**Results:**

Across the 27 countries 235,532 stillbirths, and 752,588 neonatal, 959,393 under-five, and 60,858 maternal deaths would be averted between 2015 and 2035 by implementing the All-Included intervention package, representing 67 %, 616 %, 807 % and 101 % more lives saved, respectively, than with the MDG-focused interventions. 25 % neonatal deaths averted with the All-Included intervention package would be due to asphyxia, 42 % from prematurity and 24 % from sepsis.

**Conclusions:**

Our modelling suggests a 337 % increase in the number of lives saved, which would have enormous impacts on population health. Further research could help clarify the impacts of a comprehensive scale-up of the full range of essential MNCH interventions we have modelled.

**Electronic supplementary material:**

The online version of this article (doi:10.1186/s12889-016-3238-z) contains supplementary material, which is available to authorized users.

## Background

International action to avert maternal and child mortality was propelled with the adoption of the Millennium Development Goals (MDGs) in 2000. In particular, MDG 4 and 5 call for the reduction of child mortality and improving maternal health [1]. While progress has been made towards the achievement of both goals 4 and 5, preventable maternal and child mortality persists [1–3]. Moreover, nearly half of all under-five mortality occurs during the neonatal period [2, 4].

As the MDGs transition to the Sustainable Development Goals (SDGs) this year, the Latin American and Caribbean (LAC) region has shown progress in achieving the MDGs at the regional and national level [5]. However, preventable maternal, neonatal and child mortality persists across LAC [2, 4]. Furthermore, preventable stillbirths, which were not included in the MDGs, are occurring in large numbers, particularly in LAC [3].

Research has shown that there are efficacious, cost-effective interventions that can prevent these deaths, including stillbirths [4, 6–9]. However, these maternal, newborn and child health (MNCH) interventions are not universally included in the provision of care to women and children across the LAC region. Furthermore, inequities in access to effective MNCH interventions persist across the region, which are potentially leading to even more deaths among mothers and children [4, 10, 11].

Evidence on the potential reduction in maternal, neonatal and child mortality, and stillbirths, from various packages of interventions exists, but their implementation remains limited. We conducted an analysis with the Lives Saved Tool (LiST) to compare the number of deaths potentially averted when scaling up two different set of MNCH interventions: one narrowly restricted to interventions needed to deliver the explicit MDG targets; the second set of interventions adding additional MNCH interventions that address other known causes of maternal, neonatal and child mortality. This study aims to help inform decisions on the scope of the response needed to effectively address preventable maternal, neonatal and child deaths, and stillbirths, in LAC during the post-2015 development agenda.

## Methods

All analyses were carried out in LiST, modelling software that projects the number of deaths and lives saved with selected intervention packages being scaled up over a specified time period, using Spectrum version 5.34 [12].

We identified 28 interventions in LiST that impact maternal, neonatal and child mortality, and stillbirth, which were included in this study (Table 1). Intervention variables were defined according to the LiST manual [13]. The 27 LAC countries with panels in LiST were included in this analysis: Argentina, Bahamas, Barbados, Belize, Bolivia, Brazil, Chile, Colombia, Costa Rica, Cuba, Dominican Republic, Ecuador, El Salvador, Guatemala, Guyana, Haiti, Honduras, Jamaica, Mexico, Nicaragua, Panama, Paraguay, Peru, Suriname, Trinidad & Tobago, Uruguay, and Venezuela.

**Table 1:**
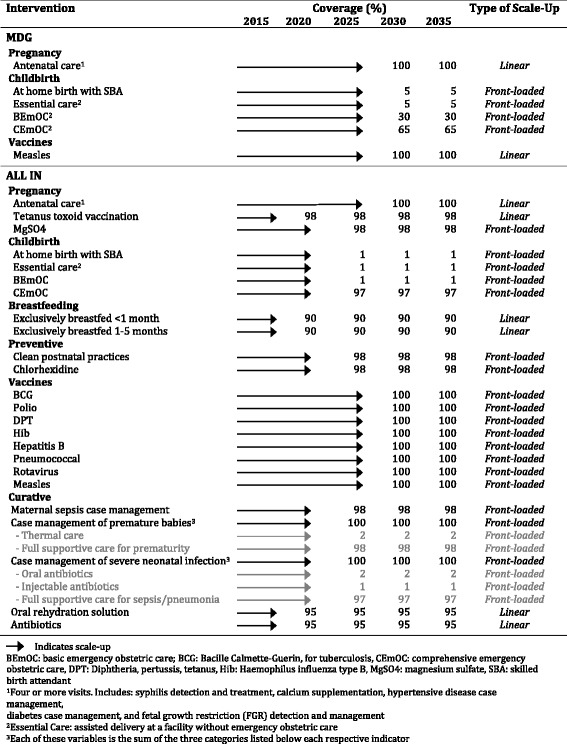
Interventions included in scale-up of service coverage intervention packages, by LiST grouping

For each country with national-level data in LiST (Belize, Bolivia, Brazil, Colombia, Costa Rica, Cuba, Dominican Republic, Ecuador, El Salvador, Guatemala, Guyana, Haiti, Honduras, Jamaica, Mexico, Nicaragua, Panama, Paraguay, Peru, Suriname, Venezuela), we verified the coverage at baseline, 2014, with the most recent national-level survey data (e.g., Multiple Indicator Cluster Surveys [MICS], Demographic and Health Surveys [DHS]), a specific dataset (e.g., immunization coverage, antibiotics for pneumonia), or based on expert opinion for each of the 28 interventions (Table 1). When no country data was available for certain interventions (Kangaroo Mother Care and the break-down of the proportion of facility deliveries at each level of care), we consulted a regional expert to determine the most accurate source for baseline coverage for each variable. For countries without national-level data in LiST (Argentina, Bahamas, Barbados, Chile, Trinidad & Tobago, Uruguay), we replaced the regionally derived estimated values in LiST with coverage estimates obtained from the most recent standardized surveys or similar global datasets [14–41], (Additional file 1). All baseline data used in this analysis is available in Additional file 2.

Three projections were used for creating three LiST scenarios for each country: baseline, MDG (basic), and All-Included (All-In). The baseline projection assumes no additional intervention scale up over the study period, bolding coverage rates constant throughout for all interventions. The MDG scenario added to the baseline scenario those interventions narrowly focused on achieving specific MDG targets, taking these to 95-100 % coverage, depending on the variable, by 2030. The All-In scenario added additional high impact interventions to the MDG scenario to address other causes of MNCH mortality (Table 1, Additional file 3). All other interventions that could be modelled within LiST were left constant. Family Planning (FP) was excluded, as Total Fertility Rates and Contraceptive Prevalence Rates in many LAC countries are close to national targets, as evidenced by an average TFR of 2.2 in LAC [42]. Our paper seeks to examine the potential impact on Neonatal, U5 and Maternal deaths in LAC that will come from expansion of non-FP but high impact interventions, going beyond the narrow set required to meet the MDGs. Nonetheless, the impact of FP in LAC, especially on adolescents, requires additional and urgent study.

While investment in the MDG or All-In intervention packages may result in investment in spillover effects that could influence the coverage rates of other interventions, there have not been sufficient scientific studies in published literature as to how to credibly estimate situations of co-coverage, or provide guidance on how to estimate their impact. Thus, our results are conservative estimates of the number of lives saved from the scale-up of each intervention package. One aim of this study is to help add to the evidence of what should be included in essential packages of health interventions, based on available data and historical scale-up trends in the region.

All three projections were from 2015 to 2035, to ensure the impact of vaccination coverage during the intervention period is included in the results of the analysis (Additional file 4). MDG (basic) and All-In packages of interventions were scaled-up between 2015 and 2030, and then kept constant between 2030 and 2035. Scale-up of each intervention was modelled either as linear, the same increase in coverage year-over-year, or front-loaded, with a rapid scale-up in the first years and a more gradual increase in coverage for the remainder of the intervention period (Table 1). This is in line with how programs in the region have traditionally scaled-up either the actual intervention being modelled, or a similar intervention. For the few countries with baseline coverage for a specific indicator greater than the MDG endline target coverage, the existing baseline coverage was held constant through 2035, for each scenario.

The MDG (basic) intervention package was the linear scale-up of: i) pregnant women receiving antenatal care to 100 %, ii) measles vaccination for children to 100 %; and iii) the front-loaded scale-up of facility-based births with access to Comprehensive Emergency Obstetric Care (CEmOC) to 65 %, facility-based births with access to Basic Emergency Obstetric Care (BEmOC) to 30 %, and assisted home deliveries to 5 % (Table 1). If delivery at home – both unassisted and assisted – was less than 5 % at baseline the proportion of home births were scaled to assisted delivery at home (range of x = 0.0 % - 4.9 %); the remaining proportion (5.0 %-x) was included in births with access to BEmOC. The basic package was purposefully designed to assess the impact of narrowly investing only in achieving and attaining MDG specific targets.

The All-In intervention scenario was built from the MDG scenario for each country, but purposively included additional interventions, available in LiST, likely to reflect the broader scope of the Sustainable Development Goals (SDGs). The scale-up, baseline and target coverage of all interventions from the MDG scenario were maintained, to ensure full comparability with the All-in Scenario. Additional interventions included in the All-In intervention package were managed as follows. There was a linear scale up, by 2020, of: tetanus toxoid vaccination for women during pregnancy to 98 %; exclusive breastfeeding for children up to six months old to 90 %; oral rehydration solution for children with diarrhea to 95 %; and antibiotics for children with pneumonia to 95 %. It also included a front-loaded scale up of the following vaccinations: Bacille Calmette-Guerin (BCG); polio; diphtheria, pertussis and tetanus (DTP); Haemophilus Influenzae Type B (Hib); Hepatitis B; pneumococcal; and rotavirus to 100 % by 2030. In addition, it incorporated the front-loaded scale-up by 2025 of: home-births with a skilled birth attendant to 1 %; essential care to 1 %; BEmOC to 1 %; and CEmOC to 97 %. The nine final interventions included for the All-In intervention were scaled-up to match the coverage figures for the delivery care type they are typically associated with. Thus, coverage with thermal care and oral antibiotics matched the sum of essential care and home-based delivery. In the same light, clean postnatal practices and Chlorhexidine (cord care) matched the sum of all three facility-based delivery options – essential care, BEmOC and CEmOC. Injectable antibiotics were added to match BEmOC coverage, while interventions included under full supportive care for sepsis/pneumonia matched CEmOC coverage. For coverage of magnesium sulfate (MgSO4), maternal sepsis case management, and full supportive care for prematurity, these were set to match the sum of BEmOC plus CEmOC coverage (Table 1).

Estimates of the total number of baseline deaths in each group annually in the LAC region were derived from published literature and interagency estimate reports [3, 43, 44].

The LiST outputs were generated using country-specific projections to determine the total number of deaths, lives saved, and mortality rates over the study period under each scenario (baseline, MDG, All-In). These outputs were categorized by intervention and by cause of death, and disaggregated by sub-population: neonates, children under five, and women. Two examples of intervention scale-up and resulting deaths averted can be found in Additional file 1. These were then aggregated for the entire 27 LAC countries studied to assess the differential impact between the scenarios on stillbirths, and on neonatal, under-five, and maternal deaths. Note that outcomes of neonates were included in the under-five group figures for lives saved, in-line with LiST convention [1, 2, 4, 5]. Lives saved, deaths and mortality rates under each scenario were projected for neonates from asphyxia, sepsis and prematurity.

## Results

**Fig. 1:**
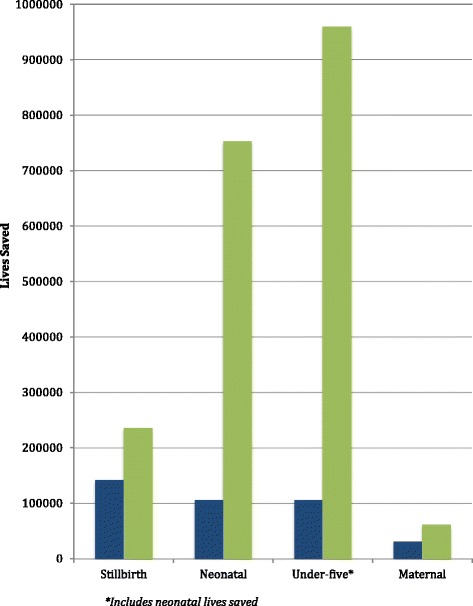
Total number of lives saved, by age group and intervention package, 2015-2035

All four groups – stillbirths, neonates, under-fives and maternal – had a significantly higher number of lives saved between 2016, the first year lives were saved from interventions scaled up in 2015, and 2035 with the All-In Intervention package, compared to the MDG intervention package. This is consistent with the reasoning for expanding the scope of the health SDGs as compared to the MDGs [45]. The MDG intervention package prevented: 141,157 stillbirths, 105,160 neonatal deaths, 105,756 under-five deaths, and 30,339 maternal deaths, between 2016 and 2035. The All-In intervention package prevented: 235,532 stillbirths, 752,588 neonatal deaths, 959,393 under-five deaths, and 60,858 maternal deaths during the same time period. The All-In scenario shows a 67 % increase in lives saved for stillbirths, 616 % for neonates, 807 % for under-fives and 101 % for mothers, compared to the MDG intervention package, reflecting the addition of high-impact interventions addressing major drivers of neonatal and maternal deaths. Approximately three-quarters (78 %) of additional under-five lives saved with the implementation of the All-In intervention package would be during the neonatal period, consistent with the distribution of deaths across the under-fives. The remaining U5 lives saved result from the increased birth cohort due to reduced neonatal mortality. This increased number of children, combined with increased coverage of other U5 interventions, leads to many more children reaching their fifth birthday compared to the narrower set of interventions under the MDG scenario (Fig. 1). We note that U5 mortality figures include ages 0 to 5 years, thus including the impact of reductions in the neonatal period (0 to 12 months of age).

Based on the average number of deaths that occurred in each of the four groups annually, between 2009-2013, the MDG intervention saved between 3.4 % and 11.0 % of deaths from stillbirth annually, and the All-In intervention package saved between 7.6 % and 16.5 % of deaths from stillbirth annually, primarily from scaling-up facility based interventions for birth and the immediate post-natal period. Between 3.5 % and 7.2 % of annual neonatal deaths would be averted with the implementation of the MDG intervention package, and between one-third (32.6 %) and nearly half (43.8 %) of annual neonatal deaths would be averted with the implementation of the All-In intervention package. The MDG package would also avert between 1.9 % and 3.6 % of under-five child deaths, as well as between 10.7 % and 22.7 % of annual maternal deaths. The All-In package would avert far greater under-five deaths – between 22.8 % and 28.0 % of all under-five deaths annually – and between one-third (27.1 %) and two-fifths (39.1 %) of annual maternal deaths (Table 2).

**Table 2:** Number of additional lives saved and proportion of deaths averted annually, by age group and intervention package, in five-year increments

		MDG		All In	
		n	(%)^a^	n	(%)^a^
Stillbirth	2020	3,320	(3.4)	7,384	(7.6)
	2025	7,858	(8.1)	13,992	(14.5)
	2030	10,702	(11.0)	15,969	(16.5)
	2035	10,444	(10.8)	15,583	(16.1)
Neonatal	2020	3,505	(3.5)	32,867	(32.6)
	2025	5,738	(5.7)	45,059	(44.7)
	2030	7,242	(7.2)	44,162	(43.8)
	2035	7,145	(7.1)	43,177	(42.8)
Under-five	2020	3,679	(1.9)	44,611	(22.8)
	2025	5,762	(2.9)	56,463	(29.0)
	2030	7,108	(3.6)	54,924	(28.0)
	2035	6,999	(3.6)	54,145	(27.6)
Maternal	2020	991	(10.7)	2,522	(27.1)
	2025	1,643	(17.7)	3,632	(39.1)
	2030	2,109	(22.7)	3,615	(38.9)
	2035	2,082	(22.4)	3,565	(38.3)

^a^Denominator is the average number of deaths that occurred annually between 2009-2013

**Fig. 2:**
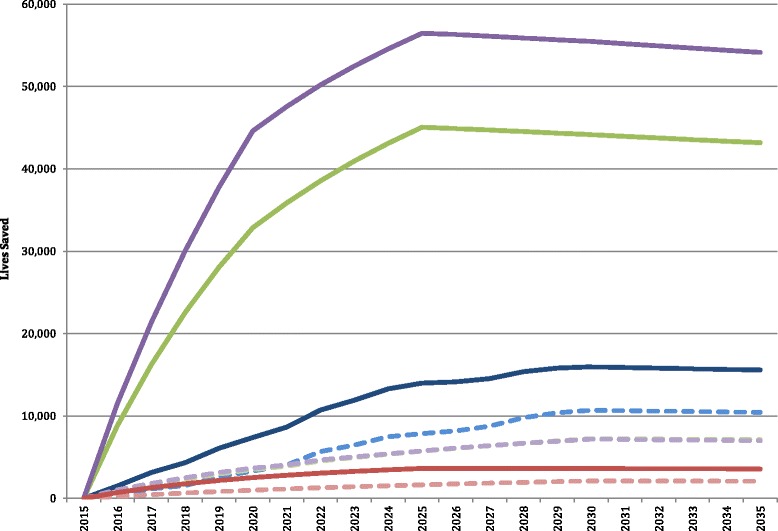
Number of lives saved annually, by age group and intervention package, 2015-2035

The All-In intervention package resulted in a substantive increase in the number of lives saved year-over-year in each age group compared the MDG intervention package. The number of stillbirths averted and neonatal, under-five and maternal lives saved annually, compared to the MDG package, rose progressively. Lives saved annually increased from 22,636 in 2016 to 116,470 in 2035 with the implementation of the All-In intervention package. This represents a 415 % increase in the number of lives saved in 2035 by the All-In intervention package, compared to the MDG intervention package (Fig. 2).

### Neonatal asphyxia, sepsis and prematurity

Tens-of-thousands of neonates would be saved from sepsis, asphyxia and prematurity with the scale-up of the MDG intervention package. The number of neonatal lives saved from asphyxia annually, compared to baseline, rose progressively from 613 in 2016 to 5,049 in 2035, saving 73,843 neonates from asphyxia between 2016 and 2035. For sepsis, implementation of the MDG intervention package resulted in the number of neonatal lives saved rising from 92 in 2016, to 697 in 2035, saving 11,798 neonates over 20 years. For prematurity, the MDG intervention package saved 170 neonatal lives in 2016, 697 in 2035, and a total of 11,798 neonatal lives over the twenty year period.

**Fig. 3:**
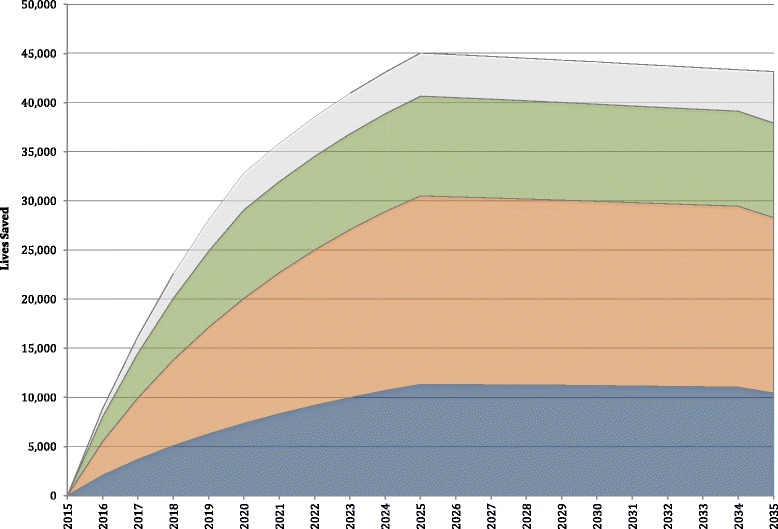
Number of neonatal lives saved annually, and from asphyxia, sepsis and prematurity, with the All In intervention package, 2015-2035

185,365, 313,423 and 177,302 neonatal deaths from asphyxia, prematurity and sepsis, respectively, would be prevented with the implementation of the All-In scale-up of interventions across the LAC region from 2016 to 2035 (Fig. 3). This represents a drastic increase in the number of neonatal lives saved from each of these three causes, compared to the MDG intervention package.

Nearly half (42 %) of all neonatal deaths prevented with the All-In intervention package would have been deaths attributed to prematurity. Another quarter (25 %) of lives saved would have been lost to asphyxia, and another quarter (24 %) of neonatal lives potentially saved would have been saved from sepsis. Together, over half a million neonatal lives in LAC would be saved from prematurity, sepsis and asphyxia over the next 20 years with the implementation of the All-In intervention package. Of all the neonatal lives saved with the All-In intervention package between 2016 and 2035, 90 % of neonatal lives saved would be saved from asphyxia, sepsis and prematurity.

## Discussion

In this paper, we present the potential impact on maternal, newborn and child mortality of two sets of high-impact interventions for which sufficient evidence exists to model them using the LiST. One set represents achieving universal health coverage (UHC) with a basic set of interventions representing those specifically included in the MDGs. A second set was designed to represent nearly universal coverage of a comprehensive package of MNCH interventions proven to save lives.

The All-In intervention package analyzed would save the lives of over an additional 1.6 million mothers and children across the LAC region over the next 20 years, compared to the MDG scenario. While the MDG indicators were selected because they have traditionally been used for proxies of additional indicators, the evidence-based interventions included in the All-In package represent a holistic package of the continuum of care for a mother, newborn and young child [4, 7, 9, 46–49]. Our results show that each LAC country would prevent significant numbers of stillbirths, and greatly reduce maternal, newborn and child deaths with this comprehensive scale-up of basic healthcare services before, during and after pregnancy.

There is much left to be done to avert preventable stillbirths in the LAC region. Increased access to ANC, facility-based delivery, and emergency obstetric and neonatal interventions has been shown to dramatically reduce stillbirths [6–8, 49]. This is consistent with our modelling, which shows that the All-In intervention package averted 235,532 stillbirths, representing a 67 % increase in stillbirths prevented compared to the limited package of MDG interventions.

The MDG intervention package also averted many deaths, saving 60 %, 14 % and 50 % neonatal, under-five and maternal lives, respectively, of the number of lives saved by the All-In intervention package. The lower impact made by the MDG intervention package may be due to the much greater coverage of comprehensive emergency obstetric and neonatal care, scale-up of exclusive breastfeeding, increased vaccination coverage, and more complete management of complications for the mother and neonate in the All In scenario [4, 9, 48, 49].

Over the 20-year interval would be maternal lives, of which the All-In intervention package was projected to save 425 % more lives than the MDG only scenario. This increase is linked to the scaling up of MgSO4 during antenatal care, access to emergency obstetric care, and maternal sepsis case management in the All-In scenario, consistent with recent studies showing their effectives in reducing maternal deaths [4, 9, 49]. Half – 50 % – maternal lives saved by intervention scale-up through 2035 are due to implementation of the All-In scenario, demonstrating the need to maintain high coverage of these vital interventions to sustainably improve the health outcomes of mothers.

Measles coverage at baseline was already 90 % – 99 % in all countries, resulting in few additional lives saved in children between 1 and 5 years old by the MDG intervention package [14]. Nearly one million lives of children under five years old would be saved in the LAC region with the implementation of the All-In intervention package. In just the first year of implementation of the All-In package, over 11,500 children’s lives would be saved, and by 2030 over 55,000 children’s lives would be saved annually; between 2020 and 2025, there was a 31 % increase in the number of children saved annually, increasing from 44,611 to 56,463. This is likely due to the large increase in coverage of proven MNCH interventions included in the All-In package around childbirth, including emergency obstetric and neonatal care, exclusive breastfeeding and vaccination coverage [4, 49].

Our results show that approximately 78 % of the 1 million under-five deaths prevented in LAC between 2016 and 2035 would occur during the neonatal period. This progress is attributable to increased coverage of emergency obstetric and neonatal care [4, 9, 49]. Additionally, the majority (90 %) of neonatal lives saved would be from increased access to appropriate treatment for prematurity, sepsis or asphyxia – the three largest killers of neonates in the LAC region [50].

Global and regional initiatives, such as The Every Newborn Action Plan (ENAP), aim to end preventable stillbirths and newborn deaths through progress towards achieving national indicators defined therein. For example, ≤12 neonatal deaths per 1,000 live births and ≤12 stillbirths per 1,000 total births by 2030. Implementing the interventions analyzed constitute a challenge on achieving ENAP´s goals in LAC. Implementation research analyzing coverage and bottlenecks regarding these interventions are within research priorities, in terms of guiding decision making on reducing newborn and infant mortality. This requires strengthening the evidence base for research, specifically monitoring and evaluation that aims to changes in coverage, quality and equity of care. As mentioned with regard to ENAP and other public health initiatives, care provided and scaled through these investments must be high quality in order to achieve desired outcomes. Concurrent investments in monitoring and evaluation of programs are required to assure resource investments are optimally put to use for mothers, newborns and children.

Previous analysis has demonstrated that when policy attention, investment and informed planning are paired with carefully tracked evidence-based interventions coverage of specific interventions tend to improve, resulting in better population health outcomes [51]. Monitoring and evaluating coverage and implementation of key interventions are critical for i) strengthening their implementation, ii) achieving the expected results, and iii) reduce inequalities, as has been shown in this analysis. This will take considerable focus and persistence from governmental, clinical and community personnel, particularly for interventions that have relatively low coverage at baseline, such as case management of severe neonatal infection and exclusive breastfeeding for the first six months of life.

### Limitations

The most recent national-level coverage estimates for each intervention were included in this analysis, though we note great variance in the source year for baseline coverage data. Additionally, there was inconsistency in the value of a given indicator across various survey methodologies, such as the MICS, DHS, and when compared to various sources of national administrative data.

We did not model the impact of family planning, as the focus of this study was to show the impact of expanding the set of interventions on rates of mortality, as compared to overall numbers of deaths. Research is needed to assess how increasing the use, as well as changing the mix, of contraceptives can contribute to averting deaths in LAC especially for adolescents and other high-risk groups.

The assumptions used in LiST to develop estimates of efficacy and impact are based on the latest guidance from the global Child Health Epidemiology Reference Group (CHERG). Nonetheless, the data used to develop LiST modelling assumptions suffers from a relative scarcity of LAC-specific studies on disease burden, efficacy of interventions, as well as a lack of health coverage survey data. Further research is needed to develop more LAC-specific metrics that could better account for regional demographic and epidemiological trends. These limitations were considered when evaluating the impact for each group and intervention package, and were discussed with regional experts to help interpret the outputs of the LiST in the context of the LAC region.

While our analysis clearly illustrates the improved health outcomes for mothers, newborns and children, we did not assess the resources required for each intervention package, some of which will likely require substantial investment. However, there have been increased commitments and focus on improving health outcomes for these groups at the global and regional levels, hinting at the potential for continuing and new sources of funding for such initiatives. More research on cost effectiveness and value for money are essential, to guide the progression and sequencing of systematic reforms and investments to realize universal coverage with a comprehensive package. We hope this spurs further dialogue and research to better understand the economic, institutional and political factors in each LAC country, at the national and sub-national level, so as to invest in and promote access to all lifesaving interventions.

## Conclusions

Averting preventable maternal, newborn and child deaths, and stillbirths, are a priority in the global health agenda, but gaps in sufficient investment for a comprehensive set of interventions remain. Our findings provide strong justification for investing in a broader set of effective, essential MNCH interventions across the LAC region. Further research – both empirical and operational – on effective and efficient service delivery approaches is needed to ensure these interventions are well planned, adequately and predictably resourced, while also monitored for quality, equity and impact. The return on investment in a comprehensive MNCH package is clear: thousands of lives saved and a greatly reduced burden of illness and disease, both of which contribute to healthier families, communities and countries.

## Abbreviations

BCG, Bacille Calmette-Guerin; BEmOC, Basic Emergency Obstetric Care; CEmOC, Comprehensive Emergency Obstetric Care; CHERG, Child Health Epidemiology Reference Group; DHS, Demographic and Health Surveys; DTP, Diphtheria, pertussis and tetanus; ENAP, Every Newborn Action Plan; FP, Family Planning; Hib, Haemophilus Influenzae Type B; LAC, Latin American and Caribbean; LiST, Lives Saved Tool; MDGs, Millennium Development Goals; MgSO4, Magnesium sulfate; MICS, Multiple Indicator Cluster Surveys; MNCH, Maternal, newborn and child health; SDGs, Sustainable Development Goals; UHC, Universal health coverage

## Additional files

Additional file 1: Assumptions for Baseline Data. Defines the assumptions used when creating the baseline intervention coverage levels, when not available from national data (e.g., survey, census) for any country. Assumptions were guided by the LiST manual (#13: http://www.jhsph.edu/research/centers-and-institutes/institute-for-international-programs/_documents/manuals/list_manual.pdf) and knowledge of care provision in the LAC region. For example, we assumed that facilities with BEmOC or CEmOC capabilities also have the capacity to provide full supportive care for prematurity and maternal sepsis case management. This file includes mortality rate tables, so the reader may see how the LiST model approximated the reduction in mortality among each age group in this analysis (e.g., neonatal, maternal). Furthermore, Additional files 1, 2 and 3 contains a table of country groupings, which were used for baseline coverage values when no intervention coverage value for a specific intervention in a particular country (e.g., ORS in Ecuador) was available. (DOCX 76 kb) Additional file 2: The baseline data. (XLSX 63 kb) Additional file 3: Projection Creation. Describes, in detail, how the baseline, MDG and All In projections were created for this analysis. This includes the order of the scale-up, type of scale-up (e.g., linear, front-loaded), target coverage level and target year, by intervention, for each projection. (DOCX 51 kb) Additional file 4: Analysis Period. Explained the rationale for the years used in this analysis. (DOCX 46 kb)
