# Supplementary material for: An analysis of three levels of scaled-up coverage for 28 interventions to avert stillbirths and maternal, newborn and child mortality in 27 countries in Latin America and the Caribbean with the Lives Saved Tool (LiST)
Source: BMC Public Health. 2016 Jul 22;16:613. doi: 10.1186/s12889-016-3238-z (PMC4957379; doi:10.1186/s12889-016-3238-z)
Supplement: Additional file 1: — Assumptions for Baseline Data. Defines the assumptions used when creating the baseline intervention coverage levels, when not available from national data (e.g., survey, census) for any country. Assumptions were guided by the LiST manual (#13: http://www.jhsph.edu/research/centers-and-institutes/institute-for-international-programs/_documents/manuals/list_manual.pdf) and knowledge of care provision in the LAC region. For example, we assumed that facilities with BEmOC or CEmOC capabilities also have the capacity to provide full supportive care for prematurity and maternal sepsis case management. This file includes mortality rate tables, so the reader may see how the LiST model approximated the reduction in mortality among each age group in this analysis (e.g., neonatal, maternal). Furthermore, Additional files 1, 2 and 3 contains a table of country groupings, which were used for baseline coverage values when no intervention coverage value for a specific intervention in a particular country (e.g., ORS in Ecuador) was available. (DOCX 76 kb) [file 12889_2016_3238_MOESM1_ESM.docx]

Technical Annex

**Additional file 1: Assumptions for Baseline Data**

Baseline coverage data for 27 LAC countries (Argentina, Bahamas, Barbados, Belize, Bolivia, Brazil, Chile, Colombia, Costa Rica, Cuba, Dominican Republic, Ecuador, El Salvador, Guatemala, Guyana, Haiti, Honduras, Jamaica, Mexico, Nicaragua, Panama, Paraguay, Peru, Suriname, Trinidad & Tobago, Uruguay Venezuela) was obtained through various UN agencies’ reports and national-level surveys, such as MICS and DHS. In order to move towards completing the baseline data, for the relevant variables, for these countries in LiST, certain assumptions, as well as proxy-calculations, were made, which are outlined below.

The proportion of children receiving the **BCG** vaccine in the Bahamas, Barbados, Suriname and Trinidad & Tobago is consistently listed as “not applicable” in PAHO/WHO reports on the current health situation in the Americas. Thus, the baseline coverage data for BCG vaccination in these 4 countries was set to 0%.

The LiST Manual defines **clean postnatal practices** as the “mother washes her hands frequently, where the child lives in a clean environment and no harmful practices are performed,” and **chlorhexidine** as “cord care (betadine, chlorhexidine, or another antibiotic used.” For both of these variables, proportion of facility deliveries, the sum of “essential care,” BEmOC and CEmOC coverage, was used as a proxy. This is a conservative estimate for both of these variables.

From the proportion of deliveries occurring in a facility (obtained from experts), the breakdown into **essential care**, **BEmOC** and **CEmOC** was calculated by country grouping.† Group 1 had 70% facility deliveries with access to care at the CEmOC level; Group 2 had 60%; Group 3 had 50%; Group 4 had 40%. All groups had 20% facility deliveries with access to BEmOC level care.

As suggested by the LiST manual, **MgSO4** coverage in “Pregnancy,” for the care of signs of preeclampsia, is set at the CEmOC value. For **full supportive care for sepsis/pneumonia**, CEmOC was also used as conservative proxy estimation, so the sum total of “case management of severe neonatal infection” coverage is conservatively estimated as facility based delivery coverage.

For both **thermal care**, a component of “case management of premature babies,” and **oral antibiotics**, a component of “case management of severe neonatal infection,” the essential care value was used as a proxy. Both of these types of care are the lowest form, of three tiers, of care in each of their respective groupings, and can be handled by facilities with essential care at delivery available.

For both i) **full supportive care for prematurity**, a component of “case management of premature babies,” and ii) **maternal sepsis case management**, the sum of the BEmOC and CEmOC values was used as a proxy. This type of care can be handled by facilities with the full set of BEmOC or CEmOC available at delivery.

In the specific case of ‘case management of premature babies’, the LiST tool includes the same interventions for ‘full supportive care for prematurity’ in both BEmOC and CEmOC facilities.

This means that LiST includes all elements of “full supportive care for prematurity” in the interventions provided in BEmOC and CEmOC level facilities. While CEmOC facilities provide other services not found in BEmOC ones, such as “induction of labor”, several interventions are identical in BEmOC and CEmOC sites, including ‘full supportive care for prematurity.

Case management of prematurity are composed of three linked interventions: i) thermal care, ii) Kangaroo mother care (KMC), and iii) full supportive care for prematurity (FSC). For the last 2 interventions, KMC and FSC, the only qualifier in terms of LiST’s modeling of impact is that they have to be delivered in facilities, without specifically saying in BEmOC or CEmOC facilities. In other words, the effectiveness of KMC and FSC to avert premature deaths are the same across different kinds of facilities.

In addition, the mix between BEmOC and CEmOC changes more aggressively over time in the All-in Scenario. By the end of the intervention period, the bulk of deliveries occur in CEmOC level facilities, reflecting the additional investments required in upgrading the scope of services required to manage difficult pregnancies and at risk neonates. In the MDG scenario, the proportion of BEmOC to CEmOC facilities changed based upon historical trends in the relative proportion of each level of facility in a given country.

We referenced the LiST manual which details the interventions included in the various packages used in the LiST for bundling related services (#13: http://www.jhsph.edu/research/centers-and-institutes/institute-for-international-programs/_documents/manuals/list_manual.pdf) in the manuscript.

Table ANNNEX-1 that demonstrates how coverage values and mortality rates change across all three scenarios, using Mexico and Ecuador as examples.

For **injectable antibiotics**, a component of “case management of severe neonatal infection,” the BEmOC value was used as a proxy. Injectable antibiotics are included in the middle tier, of three tiers, of levels of care for case management of severe neonatal infection. This type of care can be handled by facilities with BEmOC delivery care available.

For **Family Planning**, in the specific case of ‘case management of premature babies’, the LiST tool includes the same interventions for ‘full supportive care for prematurity’ in both BEmOC and CEmOC facilities.

This means that LiST includes all elements of “full supportive care for prematurity” in the interventions provided in BEmOC and CEmOC level facilities. While CEmOC facilities provide other services not found in BEmOC ones, such as “induction of labor”, several interventions are identical in BEmOC and CEmOC sites, including ‘full supportive care for prematurity.

Case management of prematurity are composed of three linked interventions: i) thermal care, ii) Kangaroo mother care (KMC), and iii) full supportive care for prematurity (FSC). For the last 2 interventions, KMC and FSC, the only qualifier in terms of LiST’s modelling of impact is that they have to be delivered in facilities, without specifically saying in BeMOC or CeMOC facilities. In other words, the effectiveness of KMC and FSC to avert premature deaths are the same across different kinds of facilities.

In addition, the mix between BEmOC and CEmOC changes more aggressively over time in the All-in Scenario. By the end of the intervention period, the bulk of deliveries occur in CEmOC level facilities, reflecting the additional investments required in upgrading the scope of services required to manage difficult pregnancies and at risk neonates. In the MDG scenario, the proportion of BEmOC to CEmOC facilities changed based upon historical trends in the relative proportion of each level of facility in a given country.

We referenced the LiST manual which details the interventions included in the various packages used in the LiST for bundling related services (#13: http://www.jhsph.edu/research/centers-and-institutes/institute-for-international-programs/_documents/manuals/list_manual.pdf) in the manuscript.

**Data points proxy value from an average value**

The following interventions are still missing baseline coverage data from at least one country. Below, each intervention is listed, followed by category from LiST (Periconceptual, Pregnancy, Childbirth, Breastfeeding, Preventive, Vaccines, or Curative), and any additional notes about that intervention.

INTERVENTIONS

- **ANC** – Pregnancy, 4+ ANC visits during pregnancy
- **TT** (tetanus toxoid) – Pregnancy, at least 2 doses
- **Exclusive breastfeeding <1 month** – Breastfeeding, Prevalence (as opposed to promotion). If recalculated data are not available, 0-2 months has been used as a proxy for 0-1 months
- Exclusive breastfeeding 1-5 months – Breastfeeding, Prevalence (as opposed to promotion). If recalculated data are not available, 4-5 months has been used as a proxy for 1-5 months
- **Chlorhexidine** – Preventive, Cord care with betadine, chlorhexidine, or another antibiotic
- **ORS** – Curative, For U5s. Does NOT include homemade sugar-salt solution or recommended home fluids
- **Antibiotics** – Curative, For U5s. Proportion of children 1-59 months with suspected pneumonia or ARI treated with antibiotics. Some use % given antibiotics for pneumonia as a proxy

INTERVENTION DATA POINTS

Some countries were missing baseline coverage data for select variables. The proxy coverage value for each of the following data points (excluding those with an asterix “*”) was calculated as the average baseline coverage value from countries in the same group,† as defined by regional and country experts:

- Bahamas
  - Exclusive breastfeeding <1 month
  - Exclusive breastfeeding 1-5 months
  - ORS
  - Antibiotics
- Barbados
  - TT (tetanus toxoid)
  - Exclusive breastfeeding <1 month*
  - Antibiotics
- Chile
  - ANC
  - TT (tetanus toxoid)
  - Clean postnatal practices
  - ORS
  - Antibiotics
- Costa Rica
  - Exclusive breastfeeding <1 month*
- Cuba
  - TT (tetanus toxoid)
- Ecuador
  - ORS
- Jamaica
  - Exclusive breastfeeding <1 month*
- Mexico
  - Antibiotics
- Panama
  - TT (tetanus toxoid)
  - Exclusive breastfeeding <1 month*
  - ORS
  - Antibiotics
- Trinidad & Tobago
  - ORS
- Uruguay
  - TT (tetanus toxoid)
  - Exclusive breastfeeding <1 month*
  - ORS
  - Antibiotics
- Venezuela
  - Antibiotics

*Note: The value for “exclusive breastfeeding <1 month” was conservatively estimated as the coverage for “exclusive breastfeeding <6 months” in each respective country

† Country groupings:

| **Chile** | 1 |
| --- | --- |
| **Costa Rica** | 1 |
| **Cuba** | 1 |
| **Uruguay** | 1 |
| **Argentina** | 2 |
| **Barbados** | 2 |
| **Brazil** | 2 |
| **Ecuador** | 2 |
| **Mexico** | 2 |
| **Panama** | 2 |
| **Paraguay** | 2 |
| **Trinidad & Tobago** | 2 |
| **Bahamas** | 3 |
| **Belize** | 3 |
| **Colombia** | 3 |
| **El Salvador** | 3 |
| **Guatemala** | 3 |
| **Jamaica** | 3 |
| **Peru** | 3 |
| **Suriname** | 3 |
| **Venezuela** | 3 |
| **Bolivia** | 4 |
| **Dominican Republic** | 4 |
| **Guyana** | 4 |
| **Haiti** | 4 |
| **Honduras** | 4 |
| **Nicaragua** | 4 |
