# Supplementary material for: An analysis of three levels of scaled-up coverage for 28 interventions to avert stillbirths and maternal, newborn and child mortality in 27 countries in Latin America and the Caribbean with the Lives Saved Tool (LiST)
Source: BMC Public Health. 2016 Jul 22;16:613. doi: 10.1186/s12889-016-3238-z (PMC4957379; doi:10.1186/s12889-016-3238-z)
Supplement: Additional file 3: — Projection Creation. Describes, in detail, how the baseline, MDG and All In projections were created for this analysis. This includes the order of the scale-up, type of scale-up (e.g., linear, front-loaded), target coverage level and target year, by intervention, for each projection. (DOCX 51 kb) [file 12889_2016_3238_MOESM3_ESM.docx]

**Additional file 3: Projection Creation**

Three projected were created for this project: baseline, MDGs and All In, which are detailed below. Definitions of each type of scale-up:

- Front Loaded: more rapid scale-up in the first years, with a more gradual increase in coverage later in the intervention period
- Linear: equal year-over-year increase in coverage across the intervention period

**Baseline**

1. Set “year boundaries for your projection” for:
   1. 1970 (First year), where the **projection** starts in 2015
   2. 2035 (Final year)
2. Check: DemProj, AIM, LiST & Fam Plan under “Activate modules for use in your projection”
3. Click on “Default Data”
   1. Leave “Select” as Country
   2. Highlight Country for projection
   3. Record source of data
   4. Record any notes on source of data (or lack thereof)
4. Alter “LiST Configuration”
   1. Use MICS/DHS to verify & validate baseline (2012-2015)
      1. **ANC**, **exclusive breastfeeding** (both <1 month, & 1-5months), **ORS** (U5s) & **antibiotics** (U5s)
   2. Use WHO/UNICEF best estimates to verify **immunizations**, including **TT, BCG, polio, DPT, Hib, HepB, pneumococcal, rotavirus** & **measles**
   3. For facility-based deliveries, uncheck box and enter **essential care, BEmOC** & **CEmOC** using expert’s knowledge of **facility-based** care coverage, and expert’s groupings of countries
   4. Use facility-based delivery (sum of essential care + CEmOC + BEmOC) as a proxy for **clean postnatal practices** & **chlorhexidine** (cord care)
   5. Use BEmOC + CEmOC as a proxy for **maternal sepsis case management** & **full supportive care for prematurity**
   6. Use essential care as a proxy for **thermal care** & **oral antibiotics**
   7. Use BEmOC as a proxy for **injectable antibiotics**
   8. Use CEmOC as a proxy for **MgSO4** (for pre-eclampsia) & **full supportive care for sepsis/pneumonia**

**MDGs** (additional steps) -> 2016 (First year of intervention program)

1. Front Loaded scale-up to 100% children vaccinated for **measles** by 2030, then 100% (2031-2035)
2. Linear scale-up to 100% pregnant women receiving **ANC*** by 2030, then 100% (2031-2035)
   1. *First check “*Antenatal care – automatically calculate components”, then scaleup ANC, then fix MgSO4 (so is new, corrected baseline coverage values for 2012 – 2035)*
   2. *NOTE: this means that MgSO4 for pre-eclampsia is not scaled up as ANC is scaled up*
3. Front Loaded scale-up of **facility based birth**: BEmOC 30% & CEmOC 65% (at least) by 2030, then duplicate for 5 following years (2031 – 2035)
   1. Let “assisted delivery @ home” & “unassisted” self-populate
   2. *If facility based birth is already GE 95%, do not change*
4. Front Loaded scale-up of **assisted delivery @ home** to 5% (*at most*; will be a linear scale-up to: 95% - facility based births in 2030) in 2030
   1. Constant at 5% (or less; see calculation described above) after (2031-2035)
   2. *If there is a value of unassisted deliveries at home (e.g., 0.4%), then scale those to assisted deliveries at home by 2030 (from 2016), and hold constant after (2031-2035)*

**ALL IN** (additional steps) -> 2016 (First year of intervention program)

1. Linear scale-up to 98% TT vaccination (pregnancy) by 2020
   1. Duplicate after (2020 – 2035)
2. Front Loaded scale-up to 1% home (with SBA); 1% essential, 1% BEmOC, 97% CEmOC by 2025
   1. Subtract whatever is over 100% from Essential Care
   2. Duplicate after (2025 – 2035)
3. Linear scale-up to at least 90% infants exclusively **breastfed** (ratio of 90 : 5 : 4 : 1 for exclusive through not breastfed) from 0-6 months of age by 2020
   1. Duplicate after (2020 – 2035)
4. Front Loaded scale-up to 100% children vaccinated for **BCG, polio, DTP, Hib, HepB, Pneumococcal** & **rotavirus** by 2030
   1. Duplicate after (2030 – 2035)
5. Linear scale-up of **ORS** (U5s) and **antibiotics** (U5s) to 95% by 2020
   1. Duplicate after (2020 – 2035)

*Scaled-up matching home-based, Essential Care, CEmOC and/or BEmOC:*

**Essential care + home-based:**

Thermal care (curative)

Oral antibiotics (curative)

**BEmOC:**

Injectable antibiotics (curative)

**CEmOC:**

Full supportive care for sepsis/pneumonia (curative)

**BEmOC + CEmOC:**

MgSO4 (pregnancy)

Maternal sepsis case management (curative)

Full supportive care for prematurity (curative)

**Facility-based delivery (Essential + BEmOC + CEmOC):**

Clean postnatal practices (preventive)

Chlorhexidine / cord care (preventive)

**Note: ANC = syphilis detection & treatment, calcium supplementation, hypertensive disease case management, diabetes case management, and FGR – fetal growth restriction detection & management*
