# Supplementary material for: An analysis of three levels of scaled-up coverage for 28 interventions to avert stillbirths and maternal, newborn and child mortality in 27 countries in Latin America and the Caribbean with the Lives Saved Tool (LiST)
Source: BMC Public Health. 2016 Jul 22;16:613. doi: 10.1186/s12889-016-3238-z (PMC4957379; doi:10.1186/s12889-016-3238-z)
Supplement: Additional file 4: — Analysis Period. Explained the rationale for the years used in this analysis. (DOCX 46 kb) [file 12889_2016_3238_MOESM4_ESM.docx]

**Additional file 4: Analysis Period**

The analysis period was set to match the post-2015 era of the Sustainable Development Goals (SDGs). Thus, baseline was defined as 2015, the first year of intervention was 2016 and the final year of the intervention implementation in each scenario was 2030.

Per LiST guidelines, projections were analyzed through five years after the conclusion of intervention scale-up, until 2035, to allow for the impact of vaccinations to show in the model output.
